# Mineral resources prospecting by synthetic application of TM/ETM+, Quickbird and Hyperion data in the Hatu area, West Junggar, Xinjiang, China

**DOI:** 10.1038/srep21851

**Published:** 2016-02-25

**Authors:** Lei Liu, Jun Zhou, Dong Jiang, Dafang Zhuang, Lamin R. Mansaray, Zhijun Hu, Zhengbao Ji

**Affiliations:** 1 Key Laboratory of Western Mineral Resources and Geological Engineering of Ministry of Education, School of Earth Sciences and Resources, Chang’an University, Xi’an 710054, China; 2 Lanzhou AuriferouStone Mining Services Co., Ltd., Lanzhou 730030, China; 3 Institute of Geographic Sciences and Natural Resources Research, Chinese Academy of Sciences, Beijing 100101, China; 4 Geological Brigade 7 of Xinjiang Bureau of Geology and Mineral Resources, Wusu 833000, China

## Abstract

The Hatu area, West Junggar, Xinjiang, China, is situated at a potential gold-copper mineralization zone in association with quartz veins and small granitic intrusions. In order to identify the alteration zones and mineralization occurrences in this area, the Landsat Thematic Mapper (TM) and Enhanced Thematic Mapper (ETM+), Quickbird, Hyperion data and laboratory measured spectra were combined in identifying structures, alteration zones, quartz veins and small intrusions. The hue-saturation-intensity (HSI) color model transformation was applied to transform principal component analysis (PCA) combinations from R (Red), G (Green) and B (Blue) to HSI space to enhance faults. To wipe out the interference of the noise, a method, integrating Crosta technique and anomaly-overlaying selection, was proposed and implemented. Both Jet Propulsion Laboratory Spectral Library spectra and laboratory-measured spectra, combining with matched filtering method, were used to process Hyperion data. In addition, high-resolution Quickbird data were used for unraveling the quartz veins and small intrusions along the alteration zones. The Baobei fault and a SW-NE-oriented alteration zone were identified for the first time. This study eventually led to the discovery of four weak gold-copper mineralized locations through ground inspection and brought new geological knowledge of the region’s metallogeny.

Remote sensing has been extensively used for lithological mapping and mineral exploration^1^,^2^,^3^,^4^,^5^,^6^,^7^,^8^. Different data have their own advantages and challenges. As the most commonly used data sources for geologic applications in the past three decades, the Landsat Thematic Mapper (TM) and the Enhanced Thematic Mapper (ETM+) are only suitable for regional mapping due to the limitation of their coarse spatial and spectral resolution^1^,^3^,^4^,^5^. High-resolution data, such as QuickBird and WorldView-2, provide the opportunity to outline some geologic objects, such as small intrusive bodies and minor veins, at local scale^4^,^7^. Hyperspectral imagery, especially Hyperion, provides the ability to remotely map altered zones and even basic surface mineralogy^8^,^9^,^10^,^11^,^12^. Using multispectral, high-resolution and hyperspectral data synthetically could maximize the details of the data acquired and make remote sensing-based mineral resources prospecting more viably.

However, remote sensing data are influenced by many factors such as the acquisition properties of images^13^ and the signal-to-noise ratio (SNR)^8^. The high SNR is very important for alteration mineral mapping because the result can be limited by the random noise^8^.

Unfortunately, the SNR of TM and ETM+ data is just specified from 18 to 33^14^, which is typically ranged from 28 to 41^15^. The influence of random noise is particularly obvious when using the Crosta technique for alteration mineral mapping since numerous applications showed that the altered anomalies were contained in the noisiest principal component (usually the third or fourth principal component)^1^,^3^,^4^,^16^. Trial and error procedures show that the noise is uncorrelated and randomly distributed on the image of the third or fourth principal component (PC3 or PC4)^1^,^3^,^4^,^16^. Therefore, some of the noise is enhanced as false altered anomalies which would be confused with the actual altered mineralization zones. Moreover, many random factors are known to be the important cause as well. These interference-induced false anomalies should be removed since it’s difficult to distinguish them from real anomalies.

The Western Junggar region, located between Altay Shan and Tianshan, extends westward to the Junggar-Balkhash system adjacent to Kazakhstan, and eastward to the Junggar Basin in China (Fig. 1)^17^. Carboniferous granitic intrusions are widespread throughout Western Junggar especially around the Darbut fault^17^. The structural and thermodynamic conditions show that the hanging wall and foot wall of Darbut has large mineralization potential, identified by many ore deposits (mostly gold and copper) like Hatu (quartz vein-related gold deposit)^18^,^19^,^20^,^21^, Baobei (quartz vein-related gold deposit)^22^, Baogutu (porphyry-type copper deposit with gold and molybdenum as by-products)^17^, Tuketuke (porphyry type copper deposits)^23^, Jafushaersu (porphyry type molybdenum-copper deposit)^5^ and Baobei (granite intrusion type gold-copper-molybdenum deposit)^17^,^22^. The known mineral deposits in the hanging wall of the Darbut suture are mainly distributed along the major fault zones and correlated closely with the secondary faults, quartz veins and intrusions, forming the Hatu gold-copper (with molybdenum) metallogenic belt^17^. The deposits are mainly quartz veins (such as QiII gold deposit) or intrusive bodies related (such as Baobei gold-copper-molybdenum deposit) (Fig. 1(b))^5^,^17^,^18^,^19^,^20^,^21^,^22^,^23^. The alteration types of the ore bodies mostly comprise silicification, pyritization, sericitization and propylitization^21^. The gold mineralization is most closely associated with beresitization^21^.

The Darbut fault is about 100 km long, trends in the SW-NE direction and dips toward the NW^24^. Similarly, the other two major faults, Hatu fault and Anqi fault, in the hanging wall of the Darbut suture, are both compresso-shear faults, SW-NE-orientated and dipping toward the NW (Fig. 1). Both Hatu fault and Anqi fault trend along the lithostratigraphic boundaries, whereas the secondary faults, restricted by the major faults, are mainly NW-SE-orientated and dislocate the SW-NE-orientated stratigraphic units obviously (Fig. 1(b)). Moreover, a large amount of known mineral deposits, occurrences and spots in the region are controlled by the two faults (Fig. 1(b)). The perpendicular distance between Hatu fault and Anqi fault is about 5 km, while the perpendicular distance between Anqi fault and Darbut fault is about 17 km (Fig. 1). Based on the stress field of the thrust fault formation, thrust faults formed simultaneously, parallel and with nearly equal intervals^25^. Thus, there should be one or two SW-NE-orientated major compresso-shear faults between the Anqi fault and Darbut fault (Fig. 1(b)).

This study, launched by the Department of Land and Resources of Xinjiang Uygur Autonomous Region, aims to prospect gold and copper mineralization in the Hatu area, especially in the four main regions related with granitic intrusions (Region 5, 10, 11 and 12 in Fig. 1(b)). Although Huan Jing-1A (HJ-1A) satellite Hyperspectral Imager data have been used to extract alteration information in the Hatu gold-copper zone^11^, the results were limited by the spectral range (from 460.03 nm to 951.54 nm) and the coarse spatial resolution (100 m).

The objective of this work is ore prospecting in use of several satellite data (including the TM/ETM+, Quickbird and Hyperion data) and image processing methods (e.g. hue-saturation-intensity color model transformation, Crosta technique, anomaly-overlaying selection, matched filtering) in the Hatu area, West Junggar, Xinjiang, China. Nothing was obtained in the four main regions except for two altered granitic porphyry bodies found in Region 11. However, the by-product, including the identification of the Baobei fault, relevant alteration zone and a series of mineralized quartz veins outside the four regions, brings new geological knowledge of the region’s metallogeny. It is therefore conducted to arouse further research.

## Results

### TM/ETM+ Data Processing Result

#### Structural Interpretation

ETM+ data, acquired on 19^th^ July 2002, were chosen and processed to interpret regional faults. By using the principal component analysis (PCA) and hue-saturation-intensity (HSI) color model transformation, PC1, PC2, and PC3 were transformed from R (Red), G (Green) and B (Blue) to HSI space, and the false color composite imagery of I, H, and S in RGB allowed discrimination of the major faults, secondary faults and even lineaments (Fig. 2).

**Figure 1:**
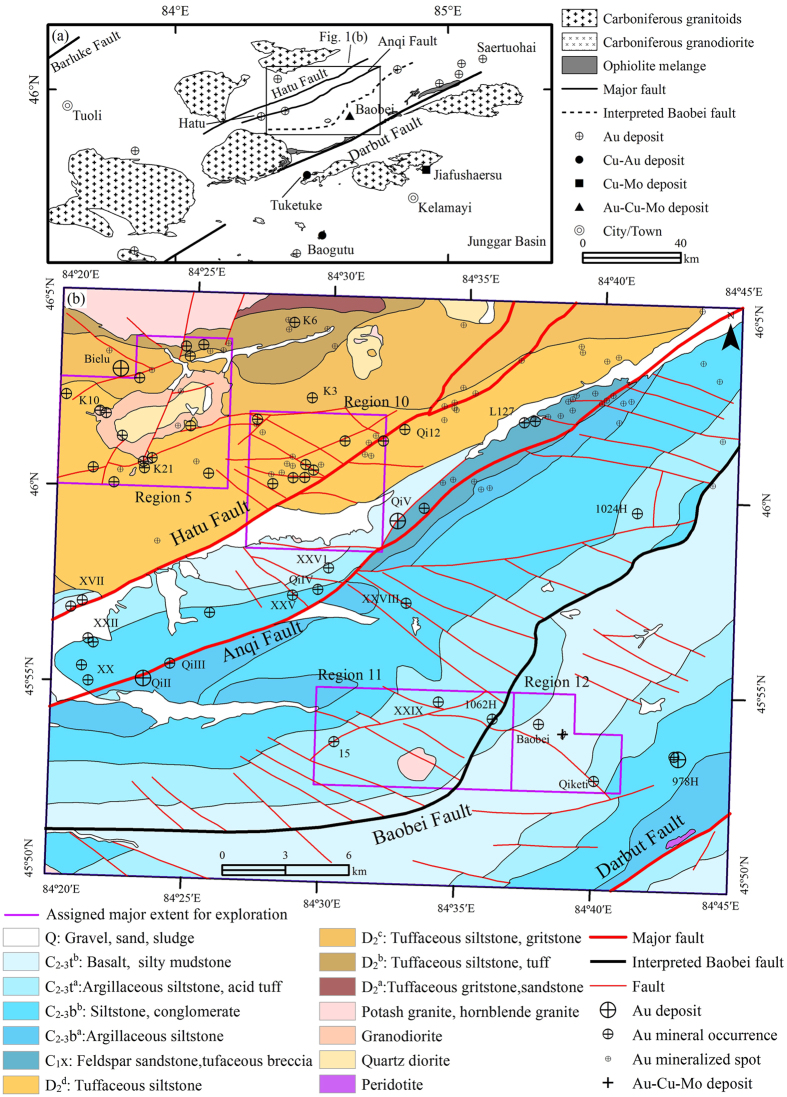
(**a**) Generalized geological map of Western Junggar, NW China, showing the distribution of the intrusions, ophiolite belts and main faults. (**b**) Geological map of the study area. Q, Quaternary; C_2-3_t^a^ and C_2-3_t^b^, Upper and Lower Tailegula Formation, Carboniferous; C_2-3_b^a^ and C_2-3_b^b^, Upper and Lower Baogutu Formation, Carboniferous; C_1_x, Xibeikulasi Formation, Lower Carboniferous; D_2_^a^, D_2_^b^, D_2_^c^, D_2_^d^, different stratigraphic units of Middle Devonian. Au, gold; Cu, copper; Mo, molybdenum. The map was generated using ArcMap v10.2 (http://www.esri.com/software/arcgis/arcgis-for-desktop) on the basis of field inspection and remote sensing data.

**Figure 2:**
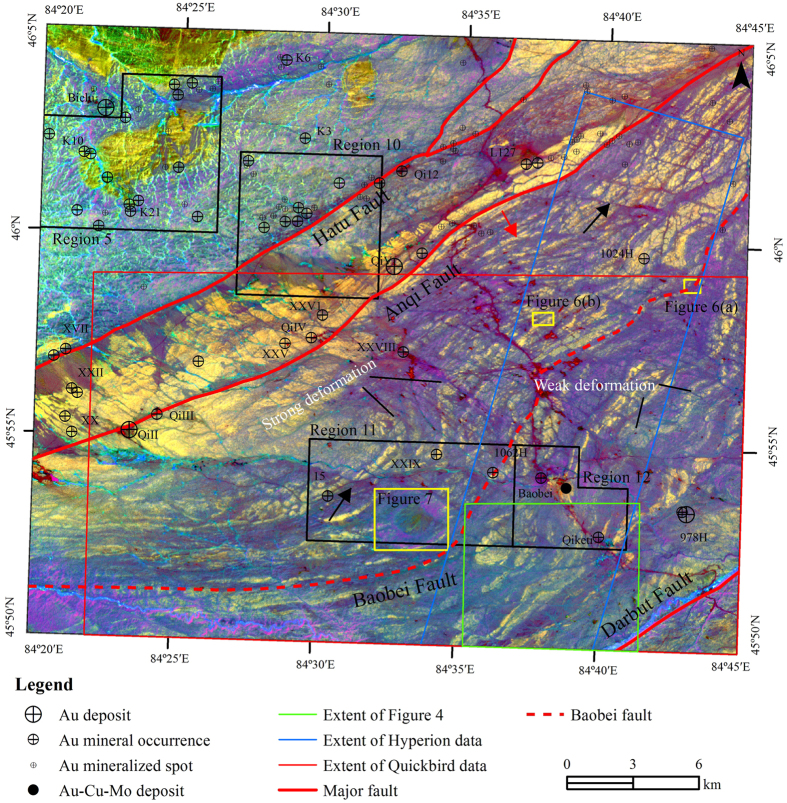
PC1, PC2 and PC3 transformed to HSI space with I, H and S in RGB image. The black arrows point to two of the NW-SE-oriented secondary faults. The red arrow points to a typical SW-NE-oriented lineament. The map was generated using ArcMap v10.2 (http://www.esri.com/software/arcgis/arcgis-for-desktop) on the basis of ETM+ data courtesy of the U.S. Geological Survey (http://landsat.usgs.gov). Figure 2 shows the lineaments of study area, mainly SW-NE-oriented, were clearly extracted in the HSI image which could reflect the strike, extension and flexure of the strata (Tailegula and Baogutu Formation). ETM+ data, acquired on 19^th^ July 2002, were chosen and processed to interpret regional faults. By using the PCA and HSI color model transformation, PC1, PC2, and PC3 were transformed from RGB to HSI space, and the false color composite imagery of I, H, and S in RGB allowed discrimination of the major faults, secondary faults and even lineaments. The major faults, Hatu fault, Anqi fault and Darbut fault, were recognized based on the differences of tone and texture between their hanging walls and footwalls. Moreover, another SW-NE-oriented major fault, named Baobei fault, was interpreted for the first time.

The lineaments, mainly SW-NE-oriented, were clearly extracted in the HSI image which could reflect the strike, extension and flexure of the strata (Tailegula and Baogutu Formation). A series of NW-SE-oriented secondary faults have been interpreted, most of which dislocate the SW-NE-oriented stratigraphic units (Figs 1 and 2). Two typical secondary faults were highlighted and pointed by the black arrows in Fig. 2. The major faults, Hatu fault, Anqi fault and Darbut fault, were recognized based on the differences of tone and texture between their hanging walls and footwalls. Moreover, another SW-NE-oriented major fault, named Baobei fault, was interpreted for the first time based on the following proofs (Figs 1 and 2).

Firstly, thrust faults of the same grade are formed simultaneously at nearly equal intervals (perpendicular distances), based on the stress field of the thrust fault formation^25^. The distance between Anqi fault and Darbut fault is three times greater than that of the distance between Hatu fault and Anqi fault (Fig. 1). There should be at least one SW-NE-orientated major compresso-shear fault between the Anqi fault and Darbut fault.

Secondly, both Hatu fault and Anqi fault trend along the lithostratigraphic boundaries, while Baobei fault extends along the boundary between Upper and Lower Carboniferous Tailegula Formation (Fig. 1). The strata (SW-NE-orientated yellow or blue belts in Fig. 2) in the hanging wall of the Baobei fault (NW side) were identified by narrow and dense lineaments, whereas the lineaments were wide and sparse in the footwall.

Thirdly, the NW-SE-orientated secondary faults were much denser in the hanging wall than that in the footwall because most secondary faults could not pass through the SW-NE-orientated Baobei fault (Figs 1 and 2). Some of the secondary faults have been checked in the field and proved by the displacement of strata (Figs 1 and 2). On the contrary, the major faults, including Hatu fault, Anqi fault and Baobei fault, could only be discriminated by the geomorphic features in the field.

Fourthly, similar to Hatu fault and Anqi fault, some ore deposits (e.g. Baobei Au-Cu-Mo deposit, 1062H Au occurrence and 1024H Au occurrence) were controlled by the Baobei fault (Fig. 1).

Fifthly, different with Hatu fault and Anqi fault, some small granitic intrusions (Baobei, No 11 and two newly discovered intrusive bodies) were controlled by the Baobei fault. Moreover, the Baobei Au-Cu-Mo deposit occurs within the Baobei granite (Fig. 1).

Sixthly, the strata along the Baobei fault show obvious flexures. The plastic deformation illustrates the concentration of stress. However, the flexures in the hanging wall are more intense than that in the footwall (Fig. 2), which indicates the existence of displacement between the hanging wall and footwall.

**Figure 3:**
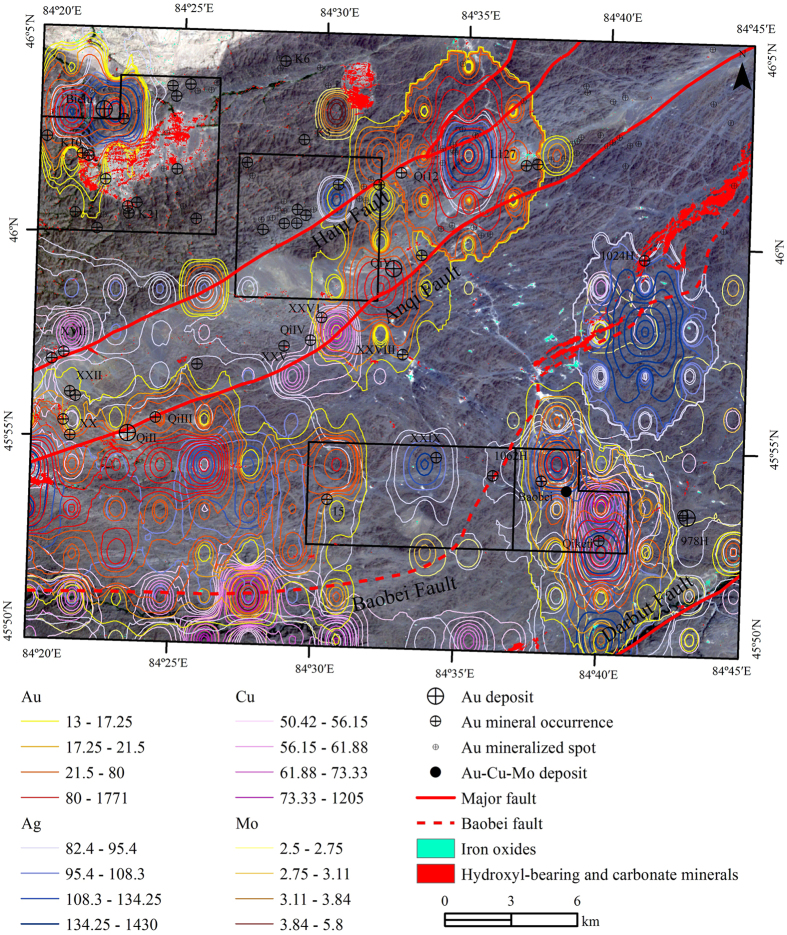
Result of hydroxyl-bearing and carbonate minerals and iron oxides extracted by using Crosta technique and 4 scene anomaly-overlaying selection. The geochemical anomalies were overlaid on the remote sensing image for synthetic analysis. The map was generated using ArcMap v10.2 (http://www.esri.com/software/arcgis/arcgis-for-desktop) on the basis of TM and ETM+ data courtesy of the U.S. Geological Survey (http://landsat.usgs.gov).

Seventhly, a new alteration zone, SW-NE-oriented and extended for more than 20 km, was identified along the Baobei fault, which could be another proof for the existence of the fault (Fig. 3).

Finally, the geochemical anomalies of the six elements, Au, Ag, Cu, Mo, As and Sb, were significantly controlled by the SW-NE-oriented major faults (Fig. 3). In particular, the anomalies of Cu and Mo were closely controlled by the Baobei fault and clustered along the Baobei fault (Fig. 3).

#### Anomaly-Overlaying Selection-Based Alteration Mineral Mapping

**Figure 4:**
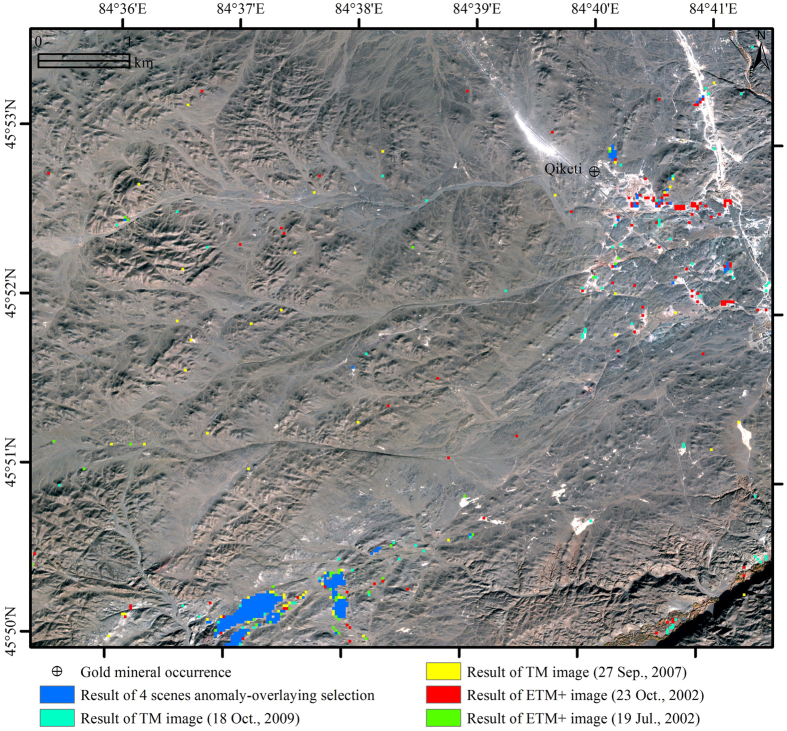
Four single results of hydroxyl-bearing and carbonate minerals extracted by using Crosta technique and the result of 4 scenes anomaly-overlaying selection in the south of the study area. Many punctate anomalies distributed randomly and independently on the image (as red, cyan, green and yellow pixels), were considered as noise-induced anomalies and should be wiped out. On the contrary, the pixels in blue color represented pixels that were identified as anomalies on more than 2 of the 4 results. After using the anomaly-overlaying selection method, all of the noise-induced anomalies have been wiped out. The map was generated using ArcMap v10.2 (http://www.esri.com/software/arcgis/arcgis-for-desktop) on the basis of TM and ETM+ data courtesy of the U.S. Geological Survey (http://landsat.usgs.gov).

To show the influence of random noise and other interferences, four single results of hydroxyl-bearing and carbonate minerals extracted by using Crosta technique were partly enlarged and compared in Fig. 4. Figure 4 illustrates the differences among the four results. Many punctate anomalies distributed randomly and independently on the image (as red, cyan, green and yellow pixels), were considered as interference-induced anomalies and should be wiped out. On the contrary, the pixels in blue color represented pixels that were identified as anomalies on more than 2 of the 4 results. After using the anomaly-overlaying selection method, all of the interference-induced anomalies have been removed (Fig. 4). Figure 3 was the final result of alteration mineral mapping using Crosta technique and anomaly-overlaying selection method.

The iron oxides mainly occur in the granitic bodies (yellow color) and agree well with some deposits such as Baobei gold-copper-molybdenum deposit, L127 gold deposit (Fig. 3). All of the hydroxyls or carbonate minerals are located in the proximity of granitic intrusions, major faults and secondary faults. Some of the alteration zones agreed very well with the known deposits in the study area. A new alteration zone, SW-NE-oriented, was identified along the Baobei fault, which is more than 20 km in length and 1 km in width (Fig. 3).

### Hyperion Data Processing

**Figure 5:**
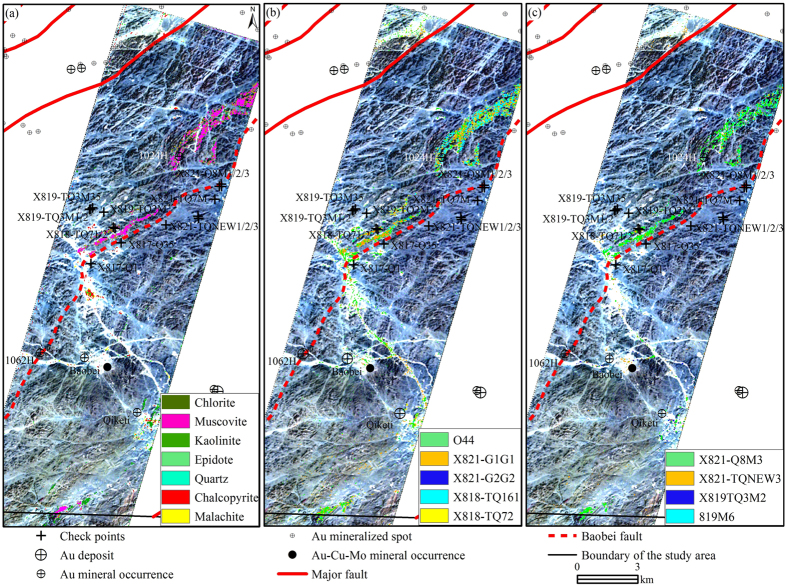
Alteration minerals extracted from Hyperion data by using matched filtering. (**a**) shows the result of Hyperion data processed using matched filtering (MF) and showing the 7 reference minerals as colored pixels that are overlaid on the natural color image background of Hyperion bands 31, 21 and 14 in RGB. Some potentially interesting areas with intensive alteration minerals are detected along the Baobei fault and in the southern parts of the scene. (**b**) shows the alteration anomaly image related to laboratory-measured reflectance spectra of 5 altered samples, (**c**) shows the alteration anomaly image related to laboratory-measured reflectance spectra of 4 altered samples. The map was generated using ArcMap v10.2 (http://www.esri.com/software/arcgis/arcgis-for-desktop) on the basis of TM and ETM+ data courtesy of the U.S. Geological Survey (http://landsat.usgs.gov).

Figure 5(a) shows the result of Hyperion data processed using matched filtering (MF) and showing the 7 reference minerals as colored pixels that are overlaid on the natural color image background of Hyperion bands 31, 21 and 14 in RGB. The threshold value of each anomaly is determined by the value μ+2*σ. Note: μ and σ represent the mean value and standard deviation of the relevant MF images, respectively. The alteration minerals are obviously associated with the Baobei fault as well as known deposits and occurrences in the Hatu area. The SW-NE-oriented alteration zone is mainly induced by minerals including muscovite, chlorite, epidote and malachite.

Some gold occurrences such as 1024H, are areas associated with muscovite, chlorite, epidote and chalcopyrite, although some of the occurrences are associated with kaolinite and chalcopyrite (Qiketi gold occurrence, Baobei gold-copper-molybdenum deposit). Some potentially interesting areas with intensive alteration minerals are detected along the Baobei fault and in the southern parts of the scene (Fig. 5(a)). The new extracted alteration zone along the Baobei fault is particularly important for its intensity and scale, hence, it can be considered for future exploration.

### Quickbird Data Enhancement and Interpretation

**Figure 6:**
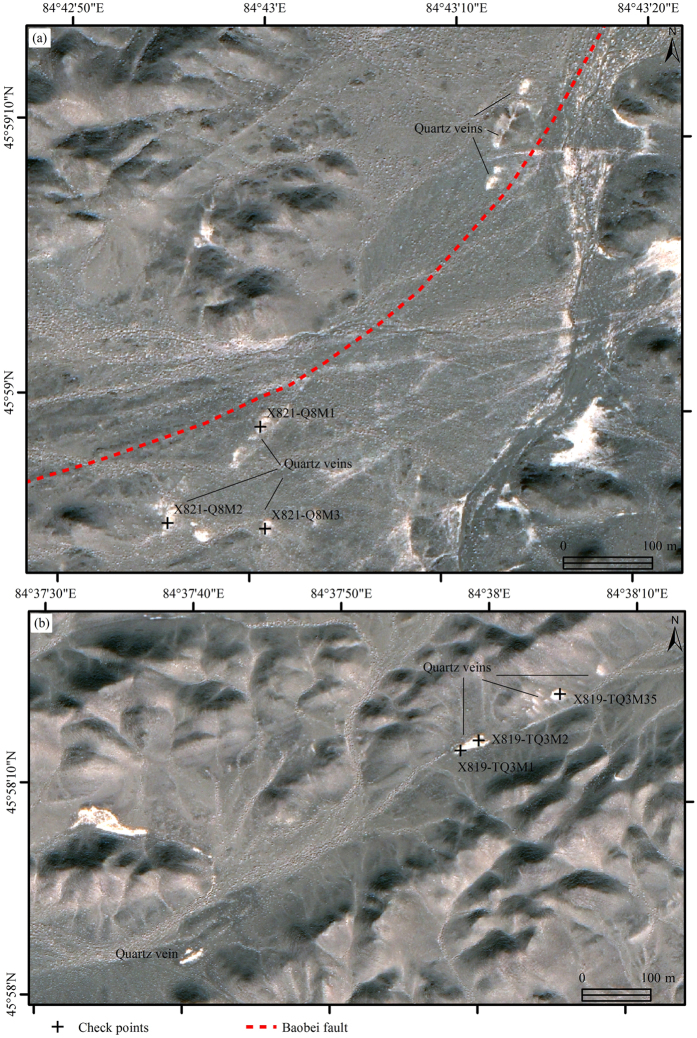
Quartz veins and their details identified using pan-sharpened Quickbird imagery bands 3, 2 and 1 in RGB (confirmed *in situ*). Figure 6 shows a series of quartz veins which were interpreted using pan-sharpened Quickbird data along the newly identified Baobei fault and alteration zone. Most of the quartz veins were 0.5 m to 3 m wide and 15 m to 100 m long. Some of the veins were covered by gravel and exposed intermittently. The map was generated using ArcMap v10.2 (http://www.esri.com/software/arcgis/arcgis-for-desktop)on the basis of Quickbird data (http://www.satimagingcorp.com/).

**Figure 7:**
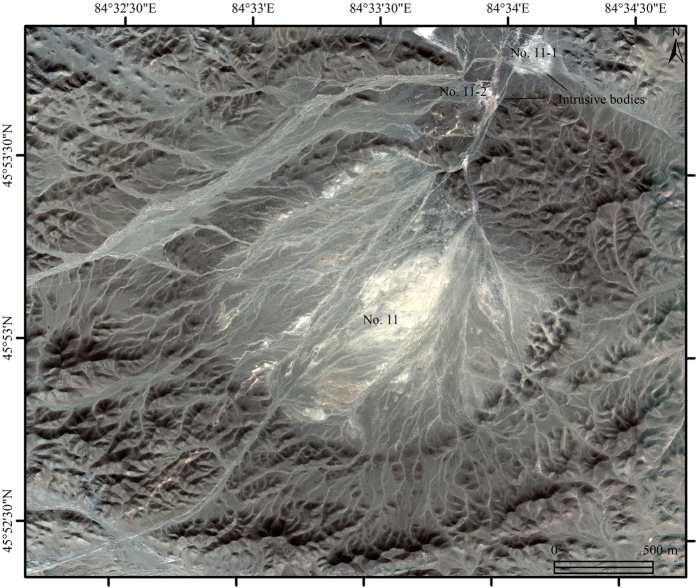
Two granitic intrusive bodies and their details identified using pan-sharpened Quickbird imagery bands 3, 2 and 1 in RGB (confirmed *in situ*). The map was generated using ArcMap v10.2 (http://www.esri.com/software/arcgis/arcgis-for-desktop) on the basis of Quickbird data (http://www.satimagingcorp.com/).

A series of quartz veins were interpreted using pan-sharpened Quickbird data along the newly identified Baobei fault and alteration zone. Most of the quartz veins were 0.5 m to 3 m wide and 15 m to 100 m long. Some of the veins were covered by gravel and exposed intermittently (Fig. 6). As mentioned before, most of the gold or copper deposits and occurrences are associated with the quartz veins, hence, newly identified alteration zones and relevant quartz veins should be considered as major targets for exploration. Moreover, two new granitic intrusive bodies in the northeast side of the No. 11 intrusive body, were interpreted for the first time (Fig. 7).

### Field Inspection and Further Studies

The alteration zone, extended along the Baobei fault for more than 20 km, has been identified by both TM/ETM+ data and Hyperion data using different methods (Figs 3 and 5). Moreover, six elements (Au, Ag, Cu, Mo, As and Sb) show anomalies clustered along the Baobei fault, which coincides with the alteration zone extracted from remote sensing data (Fig. 3). The deposits and occurrences in the Hatu area are mainly quartz vein-type or intrusive bodies related, hence, the quartz veins and intrusive bodies correlated with the Baobei fault, alteration zone and geochemical anomalies should be considered as major targets for field inspection.

To verify the results, field reconnaissance was carried out between July and August 2014. Based on the field work, the wall rock of the alteration zone was identified as greyish-green tuffaceous siltstone and mudstone with altered andesite, while the alteration was mainly composed of chloritization, sericitization, kaolinization and silicification.

The two newly identified intrusive bodies, fifteen quartz veins and some secondary faults were checked in the field (Figs 6 and 7). Both of the two intrusive bodies (No. 11-1 and 11-2), located in the major region 11, have ring-shaped outcrop and less than 150 m diameters. The two intrusive bodies were defined as granite porphyry with weak alteration (sericitization, chloritization and silicification). However, no metal mineralization has been found in the two intrusive bodies at this stage. Therefore, nothing was obtained in the four main regions except for two altered granitic porphyry bodies.

The quartz veins distributed along the Baobei fault and alteration zone were proven to be altered universally. Malachite and chalcopyrite have been observed in some quartz veins.

**Figure 8:**
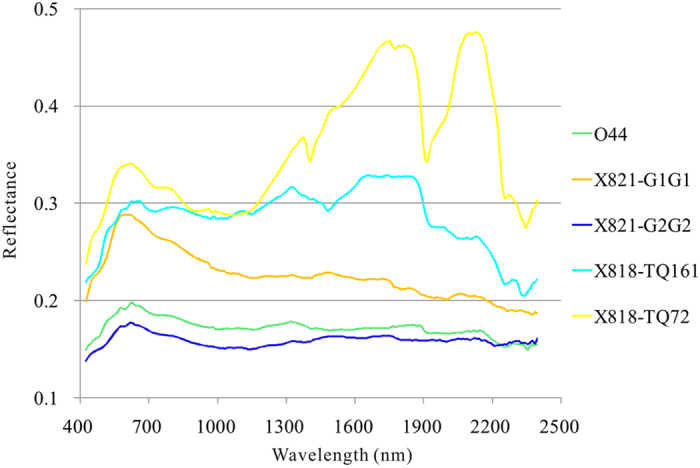
Laboratory-measured reflectance spectra of 5 altered samples in the study area. O44, weak altered wall rock (tuffaceous siltstone); X821-G1G1, greyish-green granite porphyry with weak sericitization, chloritization and silicification; X821-G2G2, dark granite porphyry with weak sericitization, chloritization and silicification; X818-TQ161, wall rock with moderate silicification, chloritization, sericitization and epidotization; X818-TQ72, strong silicification, chloritization and sericitization near the quartz vein.

**Figure 9:**
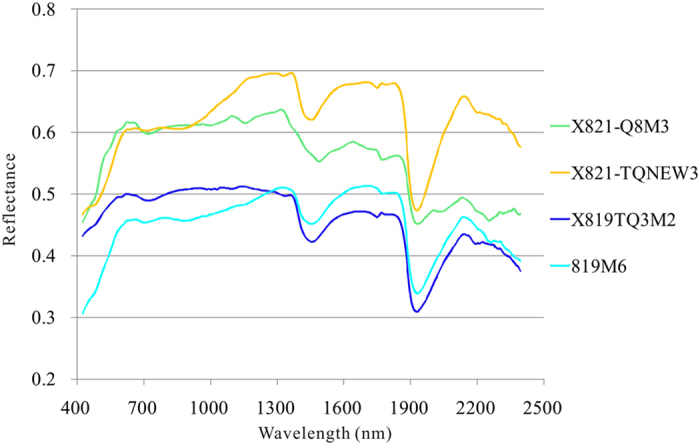
Laboratory-measured reflectance spectra of 4 samples of typical minerals in the study area. X821-Q8M3, quartz vein with malachite; X821-TQNEW3, quartz vein with chalcopyrite; X819TQ3M2, offwhite quartz vein; 819M6, weathered quartz vein.

Some samples were collected from the intrusive bodies, quartz veins and alteration zones. Fifteen selected rock samples were measured for reflectance spectra in the laboratory using an ASD FieldSpec spectrometer. All the rock samples comprise many minerals and consequently show synthetic spectral features of these minerals. The average spectra were categorized into two groups and only nine typical spectra were retained and analyzed (Figs 8 and 9).

The first group is composed of weak altered granite porphyry with different colors (X821-G1G1 and X821-G2G2), weak altered wall rock (O44), moderate altered wall rock (X818-TQ161) and strong altered rock near the quartz vein (X818-TQ72). All the five samples exhibit strong absorption features in the shortwave infrared (SWIR), centered at 2200 and 2350 nm, due to the different level of alteration including sericitization, chloritization and silicification (Fig. 8)^26^.

The second group is composed of four types of samples, quartz vein with malachite (X821-Q8M3), quartz vein with chalcopyrite (X821-TQNEW3), fresh offwhite quartz vein (X819TQ3M2) and weathered quartz vein (819M6) (Fig. 8). Except for the spectral features induced by the water molecules in minerals and rocks near 1400 nm and 1900 nm, the sample named X821-Q8M3 exhibit a moderate absorption feature in the SWIR, centered at 2250 nm, typically caused by malachite (Fig. 9)^26^.

MF method was applied to Hyperion reflectance data for all the 198 bands using the two laboratory-measured spectral groups as reference spectra shown in Figs 8 and 9. The alteration zones have nearly the same trend with the mapping result referenced by the JPL Spectra Library (Fig. 5(a–c)). The results show that the alteration near the Baobei fault is mainly sericitization, chloritization and silicification, while the mineralization is mostly associated with malachite (Table 1, Fig. 5(b,c)).

In addition, fifteen selected samples were analyzed for gold (Au) using atomic absorption spectrum (AAS) at the Xi’an Mineral Resources Supervision Center,Chinese Ministry of Lands and Resources, six of which were analyzed for copper (Cu) using inductively coupled plasma (ICP) emission spectroscopy. According to the results, four samples (X819-TQ3M2, X821-Q8M3, X821-Q8M1 and X821_TQNEW3) have shown weak enrichment of Cu, one of which has accompanied with weak enrichment of Au (X821-Q8M1) (Table 1). However, considering the large scale of the Baobei fault, alteration zone, geochemical anomalies and the large amount of quartz veins, it’s worth carrying out reconnaissance over the extent covering the zone. Limited by the coverage of Quickbird data, only a small part of the zone was dealt with in this research.

## Discussion

Different remote sensing data have their own advantages and challenges. TM and ETM+ data are suitable for regional geologic and alteration mineral mapping due to the coarse resolution^3^,^4^. Owing to lack of shortwave infrared channels, high-resolution data, such as QuickBird and WorldView-2, are commonly used in geologic objects interpretation^4^,^7^. Because of the site-specific limitations of the airborne and spaceborne hyperspectral data, they could only be applied in a limited extent at this stage^6^,^7^,^8^,^12^. Moreover, the most commonly used hyperspectral data, Hyperion, are limited by the low spatial resolution and SNR^7^,^8^. WorldView-3, launched in August 2014, has eight bands in VNIR (visible to near infrared) and an additional eight bands in the SWIR, which is expected to potentially improve geological applications of remote sensing^27^. However, there was no WorldView-3 data of the Hatu area available when the research was done.

This research has successfully enhanced the structures, identified the alteration zones, interpreted quartz veins and finally located some mineralization spots by combining TM/ETM+, Quickbird, Hyperion data with laboratory measured spectra in the Hatu gold-copper mineralization belt.

The HSI color model is a useful technique for the color enhancement of images, which allows independent control over hue, saturation, and intensity^28^. The technique, combined PCA and HSI color model transformation, has been applied for alteration mineral enhancement^29^, lithologic mapping^4^ etc. Trial and error experiments and applications have proved that the HSI color model transformation is very useful in lithology mapping and structure interpretation^4^. In this study, PC1, PC2, and PC3 of ETM+ data were transformed from RGB to HSI space, and the false color composite imagery of I, H, and S, in RGB, allowed discrimination of main structures (Fig. 2). A series of NW-SE-oriented secondary faults were identified and a SW-NE-oriented major fault, named Baobei fault, was interpreted for the first time (Fig. 2).

Satellite image always contains the interferences which could affect the understanding and interpretation of the image^30^. When processing TM/ETM+ data using Crosta technique for alteration mineral mapping, the altered anomalies are normally contained in the noisiest principal component (usually PC3 or PC4) that makes it difficult to separate real anomalies from interference-induced false anomalies^1^,^4^,^16^. Although some methods, such as adaptive fuzzy switching filter^31^ and mean filter^30^, could remove the noise from remote sensing image, these methods will remove the punctate real alteration anomalies as well. The anomaly-overlaying selection method was proposed and could effortlessly remove all the interference-induced false anomalies and retain all the real anomalies (Fig. 4).

Although the alteration zone could not be enhanced by Quickbird data, the granitic intrusive bodies and quartz veins, distributed along the Baobei fault, were accurately interpreted using the pan-sharpened Quickbird data. Because the study area is located at a potential gold-copper mineralization zone in association with quartz veins, it is important for the mineral resource exploration-orientated investigation to locate unknown quartz veins during the reconnaissance stage.

**Figure 10:**
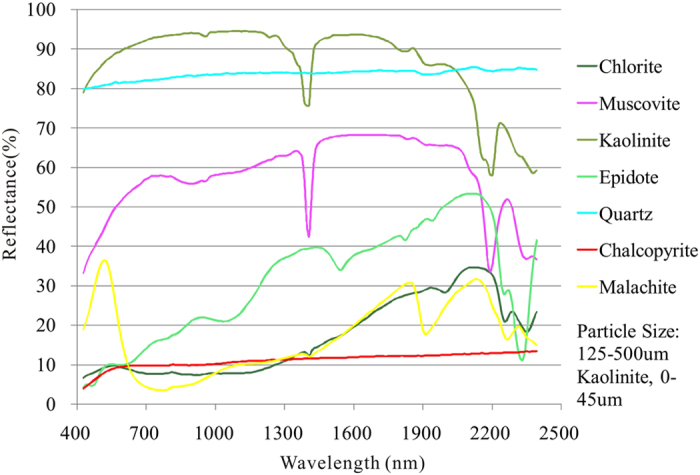
Spectroscopy data from spectral library provided by the Jet Propulsion Laboratory.

To compare the effect of using JPL Spectral Library spectra (pure minerals) and laboratory-measured spectra (altered rocks) as reference spectra, MF was applied to Hyperion reflectance data using the two types of reference spectra shown in Figs 8, 9, 10. The alteration zones showed nearly the same trend which were induced by sericitization, chloritization, silicification and malachitization (Fig. 5). The result of using laboratory-measured spectra as reference spectra was more reasonable since the altered rocks contain many rock-forming minerals and alteration minerals that form the synthetic spectra.

## Conclusions

This study demonstrates the importance and advantages of the combined use of the TM/ETM+, high-spatial resolution data and Hyperion remote sensing datasets in detecting structures, alteration zones and quartz veins associated with gold and copper mineralization, at Hatu, Xinjiang, Northwestern China. Based on the results obtained from the application of both remote sensing and geological knowledge for mineral exploration in the Hatu area, the following conclusions can be drawn.

Firstly, a series of NW-SE-oriented secondary faults have been interpreted by using the HSI color model transformation and verified in the field, which could establish the basis for understanding the regional tectonic framework.

Secondly, a new SW-NE-oriented alteration zone was extracted by using TM/ETM+ and Hyperion data that were processed by applying the anomaly-overlaying selection method and match filtering, respectively. The alteration zone is more than 20 km long, trends along the Baobei fault and could provide the macroscopic basis for the Baobei fault.

Thirdly, the quartz veins and small intrusive bodies were identified using high-resolution Quickbird data which could make the field inspection rapid and efficient. This study eventually led to the discovery of four weak gold-copper mineralized locations through the ground inspection.

Fourthly, the identification of the Baobei fault, relevant alteration zone and a series of mineralized quartz veins brings new geological knowledge of the regional metallogeny and is worthy of further and detailed research (e.g. geophysical prospecting) along this zone.

Finally, this rapid reconnaissance method by combining the TM/ETM+, Hyperion and Quickbird data is useful for mineral prospecting in the sparsely vegetated regions of Northwest China.

## Methods

### Data

Three types of satellite data, TM/ETM+, Hyperion and Quickbird, were selected in this research. Besides mineral spectra from JPL Spectral Library, laboratory-measured spectra made in the laboratory using an ASD FieldSpec spectrometer were also used to analyze the Hyperion data.

### TM/ETM+ Data

To wipe out the interference of noise, four multi-temporal TM/ETM+ images (145/27 path/row) of the study area were chosen. The two ETM+ scenes were acquired on 19^th^ July, 2002 and 23^rd^ October, 2002, while the acquisition dates of the two TM scenes were 27^th^ September, 2007 and 18^th^ October, 2009. The images were geometrically corrected by picking ground control points (GCPs) from 1:50,000 scaled topographic map sheets. The radiometric correction was implemented using the method suggested by^32^, in which calibrated Digital Numbers (DNs) were converted to Top-Of-Atmosphere (TOA) reflectance.

### Hyperion Data

The Hyperion sensor provides continuous spectral coverage of 242 bands, with 10 nm spectral resolution over the reflected spectrum from 400 to 2500 nm. Each scene covers a ground area of approximately 7.7 km in the across-track direction^8^,^10^,^33^. The dataset utilized in this study was acquired on 11^th^ October, 2013, and was subset to 198 spectral channels to exclude overlapping and unused spectral bands.

### Quickbird Data

Quickbird has four multispectral bands and a panchromatic channel covering the 450–900 nm range, with spatial resolutions of 2.44 m and 0.61 m, respectively. Quickbird data are especially useful for mapping small intrusive bodies and minor veins^4^.

Two scenes of Quickbird data, acquired on 12^th^ October, 2012 and 13^th^ October, 2012, were used for the southeast part of the study area. The images were geometrically corrected by using topographic map sheets and GPS points as reference. The multispectral images and panchromatic images were mosaicked.

### Reflectance spectra

Before field inspection, the spectra selected from JPL Spectral Library based on the alteration features were used as reference spectra to analyze the Hyperion data.

Additionally, reflectance spectra from 15 rock samples collected during field inspection were measured in the laboratory using an ASD FieldSpec with a 350 nm to 2500 nm wavelength range at 3–10-nm resolution. The purpose of the spectral measurements is to find the specific spectral features of these rock samples (instead of minerals) to aid in the evaluation of the remote sensing data. In addition, some of the rock samples were analyzed for gold and copper content in the laboratory.

### Geochemical data

Stream-sediment samples of the geochemical data were collected at a density of 1 sample per km^2^ at a scale of 1:200,000 and four samples were composited into one analytical sample for analysis of 39 elements^34^. The geochemical map was made by using Inverse Distance Weighting interpolation (IDW) method. The mean + n*standard deviation method was applied to determine the threshold values. The maps were classified by five thresholds, i.e., <μ + 1.64*σ, μ + 1.64*σ ~ μ + 2*σ, μ + 2*σ ~ μ + 3*σ, μ + 3*σ ~ μ + 4*σ and >μ + 4*σ. Note: μ and σ represent the mean value and standard deviation of the relevant geochemical data. The last four groups were treated as geochemical anomalies from weak to intense. Four major elements, Au, Ag, Cu and Mo, were analyzed with the remote sensing anomalies in this research (Fig. 4).

### Data Processing

#### TM/ETM+ Data Processing

In this study, TM/ETM+ data were used for enhancing faults and mapping regional alteration zones.

To enhance faults, the hue-saturation-intensity (HSI) color model transformation^27^ were applied to transform PCA^16^ combinations from RGB to HSI space^4^. This transformation was processed using four steps. Firstly, standard PCA was applied to the multi-spectral data of TM/ETM+ (band 1 to 5 and band 7) using the related covariance matrix^16^. Secondly, three principal components (PC1, PC2 and PC3) were selected from the matrix and placed in RGB false color composite space in order to better outline the known difference among rocks. Thirdly, the RGB false color composite was transferred to HSI space. Finally, a false color composite imagery was generated to display the HSI components by putting I, H and S in RGB^4^. Importantly, this technique is proven to be useful for distinguishing lithological characters and alteration minerals^4^.

The Crosta technique, a PCA-based method using the association of bands 1, 4, 5 and 7 for extracting hydroxyl-bearing and carbonate minerals and that of bands 1, 3, 4 and 5 for iron oxides, has been widely used in alteration mineral mapping^1^,^3^,^4^. However, the specific PCA selected for hydroxyl-bearing minerals (and carbonate) or iron oxides (usually the third or fourth principal component) is seriously affected by random noise, some of which were identified as false altered anomalies and mixed with real altered minerals. Many other random factors are known to be the important cause as well. For instance, it is common for a satellite to photograph under different natural conditions. i.e., climate, season, weather or atmosphere could be very different for the data with different acquisition dates. Furthermore, a single threshold is usually used for masking off vegetation, cloud and water in a dataset, which readily cause false anomalies, but it is impossible to eliminate those interferences completely. Needless to say, the aforementioned random disturbances are far from being constant in comparison with alteration minerals.

To wipe out the interference of the noise, an anomaly-overlaying selection method was proposed and implemented in this study. The method is based on the view that the real alteration anomalies exist on the selected PCA images derived from TM/ETM+ data with different acquisition dates, while the interferences are randomly distributed and do not coincide with those of other images (acquired at different times). The premise is that all the multi-temporal TM/ETM+ data are of high quality, have nearly the same amount of vegetation and a few clouds.

The method was implemented using four steps. Firstly, the four multi-temporal TM/ETM+ images were applied to extract the alteration minerals using the Crosta technique^1^,^3^,^4^. Secondly, all the four anomaly images for both hydroxyls and iron oxides were determined as greater than μ + 2*σ. Note: μ and σ represent the mean value and standard deviation of the relevant principal component images, respectively. Thirdly, for the overlay analysis of the four anomaly images pixel by pixel, a pixel was selected as real anomaly only when the pixel existed as anomaly on more than 2 of the 4 anomaly images. Finally, the anomaly-overlaying selected alteration mineral information was overlaid on the false color composite remote-sensing image.

### Quickbird Data Enhancement

To map small intrusive bodies and minor veins, pan-sharpened Quickbird image was created by fusing the multispectral imagery (bands 3, 2 and 1 with a spatial resolution of 2.44 m) with the 0.61 m pan imagery using the method of the Brovey Transform^35^. The Brovey Transform uses a ratio algorithm to combine the color image and high resolution data^36^.

### Hyperion Data Processing

The internal average relative reflectance (IARR) method, especially suitable for arid areas with no vegetation, was applied in this study to convert Hyperion data to relative reflectance images^37^,^38^.

Based on the alteration and mineralization characteristics of Hatu area, seven minerals, including quartz, muscovite, kaolinite, chlorite, epidote, chalcopyrite and malachite, were regarded as major alteration minerals and selected from the JPL Spectra Library (Fig. 10). Quartz has high reflectance with no diagnostic features, chalcopyrite shows low reflectance with a broad 420 nm absorption feature, while the other minerals exhibit distinct absorption features located from 2150 nm to 2400 nm with different locations and depth. Muscovite (or sericite) has a distinct Al-OH absorption feature at 2200 nm and a less intense absorption feature at 2350 nm (Fig. 10)^26^. Kaolinite exhibits Al-OH 2165 nm and 2200 nm absorption features (Fig. 10)^26^. Epidote has a distinct absorption feature at 2330 nm, while chlorite shows a distinct absorption feature at 2330 nm and a less intense absorption feature at 2220 nm. Malachite exhibits a distinct absorption at 2250 nm (Fig. 10). These spectral absorption features provide the basis for mineral extraction using remote sensing data^26^.

Matched Filtering (MF) was applied to Hyperion reflectance data for all the 198 bands using the 7 reference spectra shown in Fig. 10. MF, a partial unmixing method, maximizes the response of the known endmember and suppresses the response of the background materials by projecting each pixel vector onto a subspace, which is orthogonal to the background spectra^2^,^39^,^40^.

After synthetic analysis, field reconnaissance was carried out between July and August 2014 to verify the results, with 15 representative rock samples collected and measured for reflectance spectra in the laboratory. Furthermore, the laboratory-measured reflectance spectra were applied for processing Hyperion data to map altered zones and verify the results.

## Additional Information

**How to cite this article**: Liu, L. *et al.* Mineral resources prospecting by synthetic application of TM/ETM+, Quickbird and Hyperion data in the Hatu area, West Junggar, Xinjiang, China. *Sci. Rep.* **6**, 21851; doi: 10.1038/srep21851 (2016).

## Figures and Tables

**Table 1:** Descriptions and laboratory analysis results of field surveyed points along the alteration zone (the points described here correspond to those highlighted in Figs 5 and 6).

Sample No.	Components		Description	Alteration and mineralization
	Cu (10^−6^)	Au (10^−9^)		
X819-TQ3M2	114	6.36	The same quartz vein with X819-TQ3M1	Malachite and chalcopyrite
X821-Q8M1	132	21.0	Oily quartz vein (Fig. 7(a))	Chalcopyrite and malachite
X821-Q8M3	641	3.29	Quartz vein (Fig. 7(a))	Limonite, chalcopyrite and malachite
X821_TQNEW3	278	0.85	Oily quartz vein	Chalcopyrite and malachite

Note: ‘-’ means not being measured. Fifteen samples have been analyzed and only four major ones were listed.
